# A redox-active support for the synthesis of Au@SnO_2_ core–shell nanostructure and SnO_2_ quantum dots with efficient photoactivities†

**DOI:** 10.1039/d0ra06175a

**Published:** 2020-09-14

**Authors:** Xiaoyang Pan, Wen-Jie Chen, Huizhen Cai, Hui Li, Xue jiao Sun, Bo Weng, Zhiguo Yi

**Affiliations:** a College of Chemistry and Materials, Quanzhou Normal University Quanzhou 362000 China xypan@qztc.edu.cn; b cMACS, Department of Microbial and Molecular Systems, KU Leuven Celestijnenlaan 200F 3001 Leuven Belgium bo.weng@kuleuven.be; c State Key Lab of High Performance Ceramics and Superfine Microstructure, Shanghai Institute of Ceramics, Chinese Academy of Sciences Shanghai 200050 China zhiguo@mail.sic.ac.cn

## Abstract

A defect pyrochlore-type Sn_1.06_Nb_2_O_5.59_F_0.97_ (SnNbOF) nano-octahedron is used as a redox-active support for fabricating Au@SnO_2_ core–shell and SnO_2_ quantum dots at room temperature without the use of organic species or foreign reducing reagents. Gold (Au) and SnO_2_ components were obtained through an *in situ* redox reaction between the HAuCl_4_ and reductive Sn^2+^ ions incorporated in SnNbOF. The composition and morphology of the resulting nanocomposites (denoted as Au–SnNbOF) could be controlled by adjusting the Au/SnNbOF ratio. The Au–SnNbOF nanocomposites exhibited efficient photoactivities for methyl orange (MO) degradation under the visible light irradiation (*λ* > 420 nm), during which the MO was almost completely degraded within 8 min. Among all the samples, the 5wt% Au–SnNbOF nanocomposite had the highest rate constant (0.43 min^−1^), which was 40 times higher than that of the blank SnNbOF.

## Abstract 2

A defect pyrochlore-type Sn_1.06_Nb_2_O_5.59_F_0.97_ nano-octahedron is used as a redox-active support for the fabrication of Au@SnO_2_ core–shell structure and SnO_2_ quantum dots.

## Graphical abstract

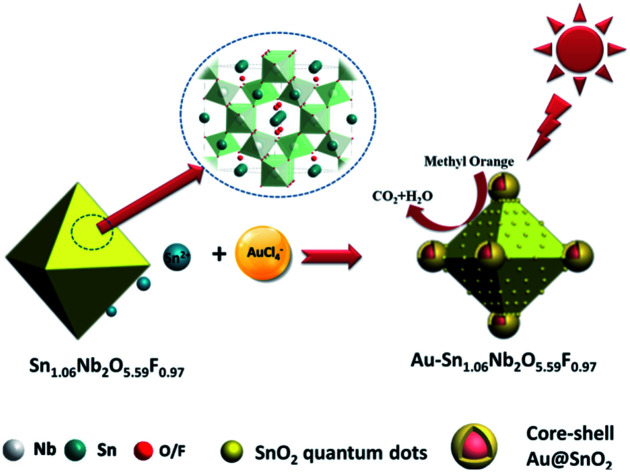

## 1 Introduction

Gold (Au) nanoparticles have been the subject of extensive research because of their unique application in many areas.^1–8^ However, when used alone, Au nanoparticles are frequently subject to agglomeration because of their high surface energy.^9–12^ To overcome this disadvantage, a plausible solution is to anchor the Au nanoparticles on specific supports.^9–12^ Among the various types of supports, metal oxides have emerged as the most promising supports, since they are abundant in nature and are frequently used in various industry applications.^9–11^

Over the years, a variety of Au-metal oxide nanocomposites have been fabricated to prevent Au from aggregation, and increased efforts have been put into the controllable synthesis of Au@metal oxide core–shell nanostructures.^6,13–20^ Encapsulation of the metal oxide shell results in the Au nanoparticles exhibiting excellent performance and prevents them from agglomeration, even under high-temperature treatment.^21,22^ However, the synthetic methods largely rely on a complicated and environmentally unfriendly procedure, which leads to the high cost of the product and environmental pollution.^6,13–20,23,24^ In view of this, it is crucial to develop a simple and green strategy for the synthesis of Au@metal oxide core–shell nanostructures.

Clearly, most of the current methods used for the synthesis of metal oxide-coated Au nanostructures require foreign reducing agents and/or organic surfactants.^1,5,25–27^ As a result, impurities are inevitably introduced, which is harmful to the performance of the Au@metal oxide.^1^ Recently, we reported a simple and green method for synthesizing the supported noble metal nanoparticles on defect pyrochlore-type Sn_1.06_Nb_2_O_5.59_F_0.97_ (SnNbOF) without the use of organic species or foreign reducing agents.^28^ On the basis of this method, we herein report the use of SnNbOF as a redox-active support for the synthesis of Au@SnO_2_ core–shell nanostructures. The successful construction of a core–shell structure is realized through an *in situ* redox reaction between HAuCl_4_ and reductive SnNbOF in an aqueous solution. Moreover, tin(iv) oxide (SnO_2_) quantum dots are formed simultaneously on the surface of the SnNbOF. The resulting nanocomposites demonstrate efficient photoactivities for methyl orange (MO) degradation.

In comparison with previous reports on the synthesis of core–shell nanostructures (Table S1†), our strategy has the following clear advantages: (i) the synthetic procedure involves one single step and requires no organic structure-directing agents; (ii) neither foreign reducing agents nor thermal treatment is necessary for the growth of the Au core and the SnO_2_ shell; (iii) SnNbOF is used as a multifunctional support-reducing agent for the Au ions, as a substrate for the growth of Au@SnO_2_ composites and, most importantly, as a structure-directing agent for a controllable synthesis of the core–shell nanostructure and (iv) the 5 wt% Au–SnNbOF nanocomposite demonstrates superior photoactivity for MO degradation, as compared with most of the photocatalysts in previous reports.^29–36^

## 2 Experimental

### 2.1. Materials

HAuCl_4_, SnF_2_, HF, NH_4_OH and Nb_2_O_5_ were purchased from Sinopharm Chemical Regent Co., Ltd. (Shanghai, China), whereas deionized water was obtained from local sources. All the materials were used as received without further purification.

### 2.2. Synthesis

Sn_1.06_Nb_2_O_5.59_F_0.97_ (SnNbOF) was synthesized according to our previous report.^28^ The growth of Au nanoparticles on the surface of the SnNbOF was conducted as follows: 0.2 g of the as-obtained SnNbOF was dispersed in 100 mL of deionized water with the aid of ultrasonication. Then, a given quantity of HAuCl_4_ in an aqueous solution was mixed with the SnNbOF suspension using vigorous stirring at room temperature. After 48 h of stirring, the final products were collected, washed with distilled water and dried at 353 K in an oven.

### 2.3. Characterization

The crystal structures of the as-prepared samples were analysed using a Rigaku Miniflex II X-ray diffractometer with Cu Kα radiation. The optical properties of the samples were characterised *via* a Cary 500 ultraviolet-visible (UV-vis) diffuse reflectance spectrophotometer (DRS), with BaSO_4_ used as the internal reflectance standard during the procedure. A field-emission scanning electron microscope (JSM-6700F) and a transmission electron microscope (TEM; JEM-2010, FEI, Tecnai G^2^ F20 FEG TEM) were used to determine the morphology and microscopic structure of the as-synthesized samples. X-ray photoelectron spectroscopy (XPS) measurements were performed using a Thermo Scientific ESCA Lab250 spectrometer consisting of a monochromatic Al Kα as the X-ray source, a hemispherical analyser and a sample stage with multi-axial adjustability to obtain the composition on the surface of the samples. All the binding energies were calibrated *via* the C 1s peak of the surface adventitious carbon at 284.6 eV. Electron Spin Resonance (ESR) signals of the radicals trapped by 5,5-dimethyl-1-pyrroline-*N*-oxide (DMPO) were recorded using a Bruker ESP300E spectrometer. Photoelectrochemical analysis was performed according to our previous report.^37^ Photoelectrochemical analysis has been performed according to our previous report.^28^ Photoluminescence analysis was carried out with a Varian Cary Eclipse spectrometer at an excitation wavelength of 325 nm.

### 2.4. Photocatalytic activities

For the photocatalytic degradation methyl orange (MO), a 30 mg photocatalyst was dispersed into 60 mL of MO solution (10 ppm) in a quartz vial. The resulting suspension was stirred in the dark for 1 h to ensure the establishment of an adsorption–desorption equilibrium between the sample and the reactant. Then, the reaction system was irradiated *via* a 300 W Xe lamp (CEL-HXF300) system with a UV-CUT filter (*λ* > 420 nm). As the reactions proceeded, 3 mL of the suspension was taken at a certain time interval and was centrifuged to remove the catalyst. Following this, the residual amount of MO in the solution was analyzed on the basis of its characteristic optical absorption at 470 nm, using a UV/vis/NIR spectrophotometer (Perkin Elmer Lambda 900) to measure the change in MO concentration with an irradiation time based on Lambert–Beer's law. The percentage of degradation is denoted as *C*/*C*_0_. Here *C* is the absorption of the MO solution at each irradiation time interval of the main peak of the absorption spectrum, whereas *C*_0_ is the absorption of the initial concentration when the absorption–desorption equilibrium was achieved.

## 3 Results and discussion

**Fig. 1:**
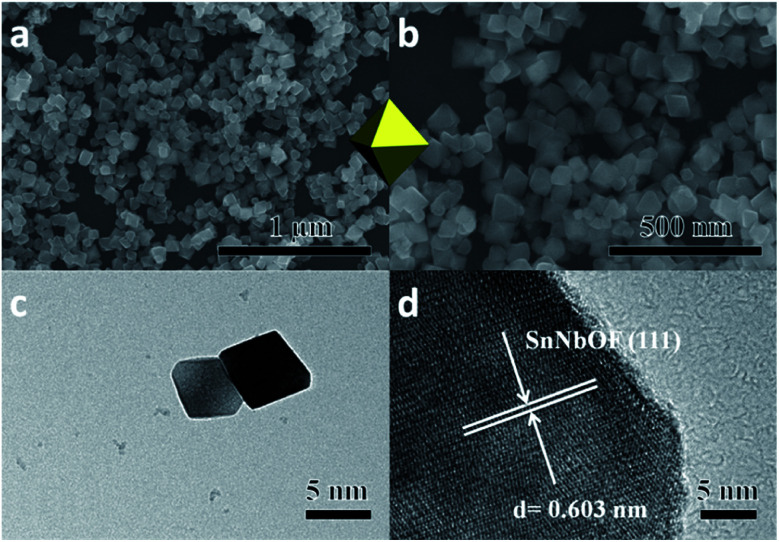
SEM (a and b), TEM (c) and HRTEM (d) images of Sn_1.06_Nb_2_O_5.59_F_0.97_ (SnNbOF).

The defect pyrochlore-type Sn_1.06_Nb_2_O_5.59_F_0.97_ (SnNbOF) was prepared *via* a hydrothermal method at 473 K for 24 h using SnF_2_ and Nb_2_O_5_·*n*H_2_O as precursors. Fig. S1† displays a typical powder X-ray diffraction of the as-obtained sample, which can be indexed to cubic structure of pyrochlore compound with cell constants of *a* = *b* = *c* = 10.557 Å.^28^ The X-ray photoelectron spectroscopy analysis revealed that the sample was composed of Sn, Nb, O and F elements (Fig. S2a†). The valence states of Sn, Nb, O and F elements were determined to be +2, +5, −2 and −1, respectively (Fig. S2b–e†). The SEM image showed that the product particles are of well-defined octahedral shape. The microstructure of SnNbOF nano-octahedron was further investigated by TEM analysis. Fig. 1c shows a low-magnification TEM image of two octahedral particles. The HRTEM image in Fig. 1d exhibits a distinct lattice spacing of 0.603 nm for the (111) plane of the SnNbOF.^28^

**Fig. 2:**
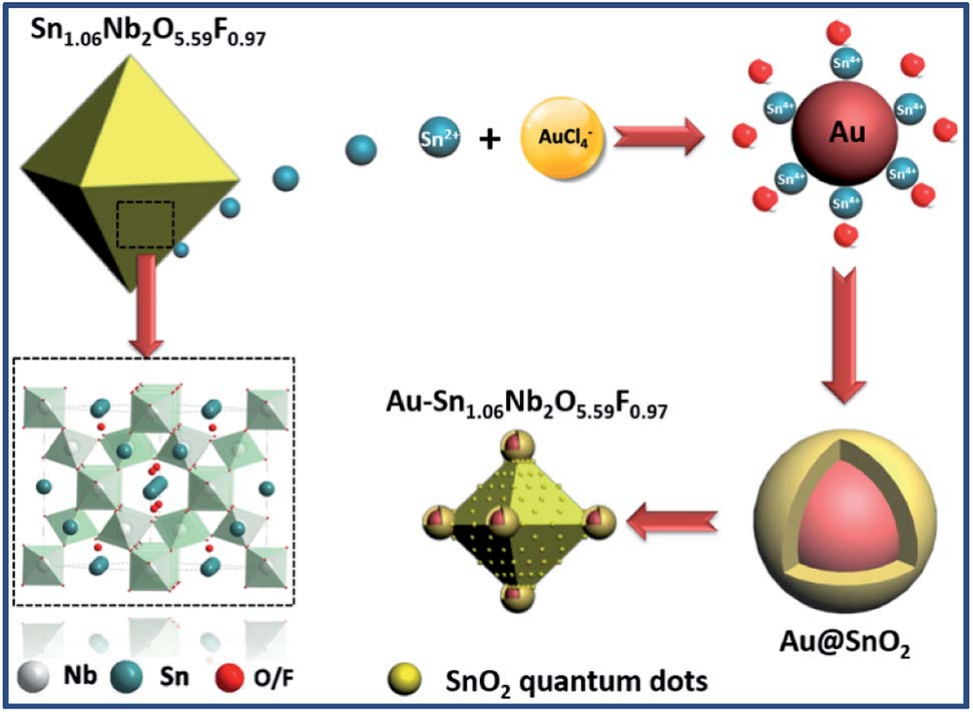
Schematic illustration of the synthetic procedure of Au@SnO_2_–SnNbOF nanocomposite.

In terms of the defect pyrochlore SnNbOF, the characteristic structural feature is a three-dimensional framework formed by octahedral NbO_6_ units (Fig. 2). The Sn^2+^ ions can move easily in the interstitial cavities within the framework.^28^ Based on these unique properties, SnNbOF could be used as a reactive support for the direct growth of Au nanoparticles *via* an *in situ* redox reaction between reductive Sn^2+^ and HAuCl_4_ in an aqueous solution (Fig. 2). The synthetic procedure was conducted at room temperature without the use of foreign reductants or organic surfactants.

The variation of the concentration of HAuCl_4_ precursors can also modulate the composition and morphology of the final product. During the synthetic procedure, a large excess of SnNbOF was used as the precursor. Therefore, the number of Au nanoparticles and Sn^4+^ ions formed (Fig. 2) depended on the Au/SnNbOF ratio. At a low ratio of 0.5 wt%, the number of Sn^4+^ ions were correspondingly low and the hydrolysis reaction of the ions barely occurred. As a result, very little SnO_2_ was formed during the synthesis. Meanwhile, at relatively high ratios (≥3 wt%), the concentration of Sn^4+^ ions was enough to induce the hydrolysis reaction. The as-formed SnO_2_ was decorated onto the surface of the Au nanoparticles and the SnNbOF. The Au@SnO_2_ core–shell nanostructure and the SnO_2_ quantum dots were subsequently obtained.

**Fig. 3:**
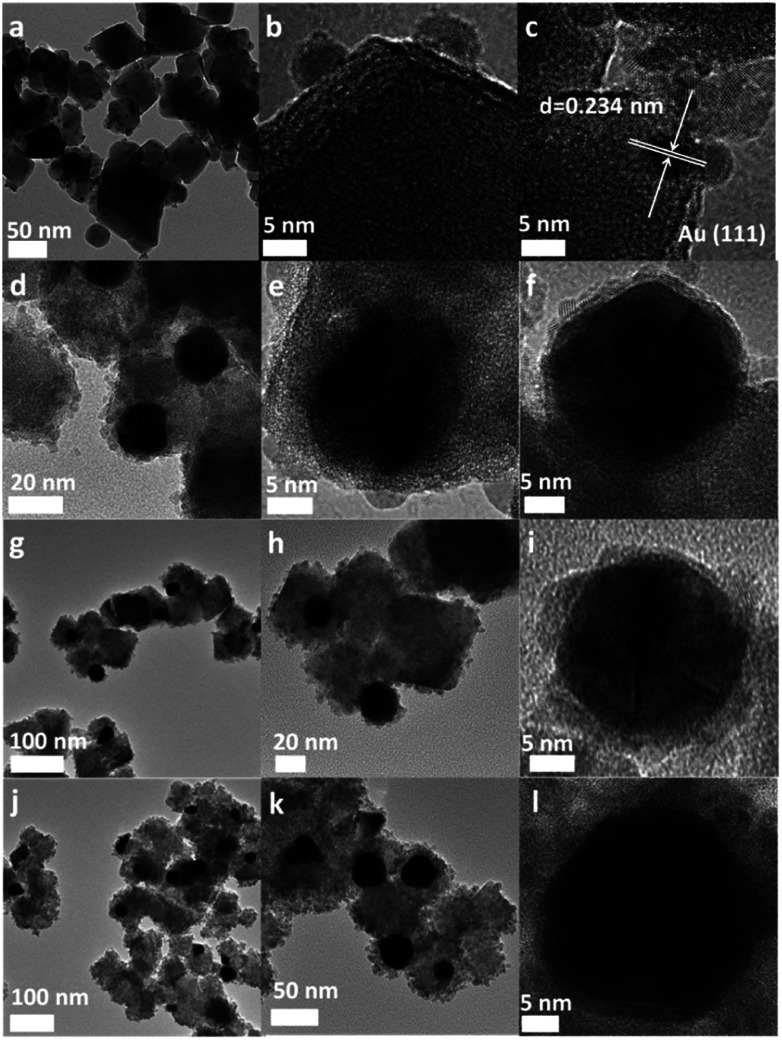
TEM and HRTEM images of 0.5 wt% Au–SnNbOF (a–c), 3 wt% Au–SnNbOF (d–f), 5 wt% Au–SnNbOF (g–i) and 10 wt% Au–SnNbOF (j–l).

The TEM observations revealed that it is possible to modulate the morphology of the nanocomposite by simply varying the Au/SnNbOF ratios (Fig. 3). For the 0.5 wt% Au-loaded SnNbOF, the resulting nanocomposite (0.5 wt% Au–SnNbOF) was composed of bare Au nanoparticles supported on the SnNbOF surface (Fig. 3a–c). No obvious SnO_2_ particles were observed on the surface of the Au particles. The increase in Au content resulted in a typical core–shell structure, in which an Au core was coated with the SnO_2_ shell (Fig. 3d–l, S3a and b†). As a result, we can obtain a surface support (0.5 wt% Au–SnNbOF) and a core–shell structure (3 wt% Au–SnNbOF, 5 wt% Au–SnNbOF and 10 wt% Au–SnNbOF), respectively.

**Fig. 4:**
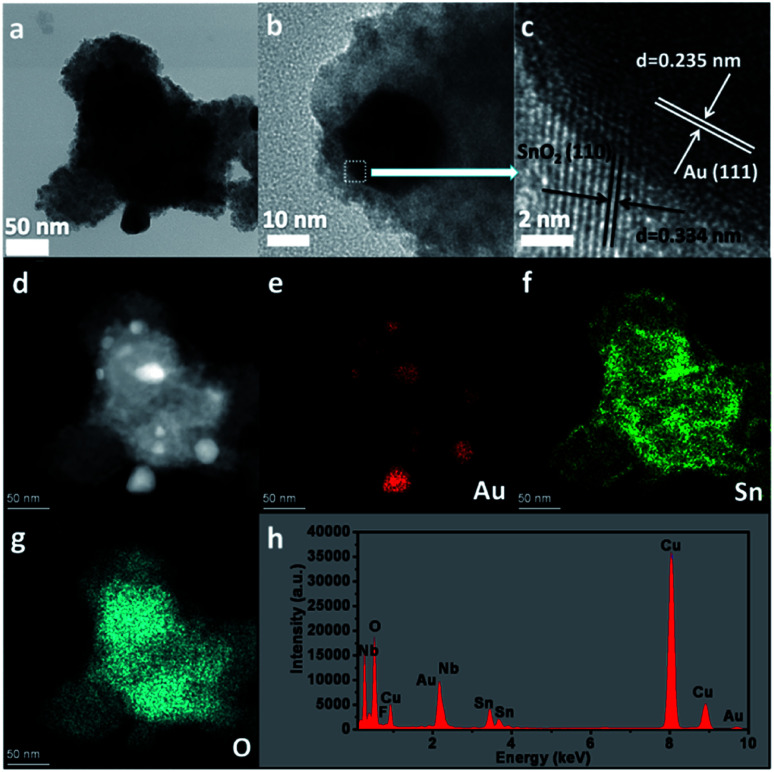
The characterizations of 10 wt% Au–SnNbOF. (a and b) TEM image, (c) HRTEM image, (d) HAADF-STEM image, (e–g) elemental mapping analysis and (h) EDX analysis.

To further investigate the core–shell structure, 10 wt% Au–SnNbOF was chosen and the nature of the nanostructure unravelled *via* high-angle annular dark-field scanning (HAADF-STEM) and elemental mapping analysis. Fig. 4a and b show the TEM images of the Au@SnO_2_ core–shell structure. The lattice spacings measured in the HRTEM image were 0.235 nm in the core and 0.334 nm in the shell (Fig. 4c), which can be ascribed to the (111) facet of the Au and the (110) plane of the SnO_2_, respectively.^6^ The HAADF-STEM image (Fig. 4d) clearly revealed that the nanocomposite had a core–shell structure, whereas the elemental mapping analysis further demonstrated that the element Au was distributed only in the core and that the Sn and O elements of the SnO_2_ were homogenously distributed throughout the whole particle (Fig. 4e–g). These results suggest that the Au cores were surrounded by SnO_2_ shells. The energy-dispersive X-ray analysis for the 10 wt% Au–SnNbOF nanocomposites also confirmed the compositions of the Au, Sn, Nb, O and F elements (Fig. 4h), suggesting that the Au@SnO_2_ composite was decorated on the SnNbOF surface. The XPS analysis revealed that Au in 10 wt% Au–SnNbOF is in the metallic state (Fig. S4a†).^28^ The coexistence of Sn(ii) and Sn(iv) in 10 wt% Au–SnNbOF suggests that some of the Sn(ii) ions in SnNbOF were oxidized by the Au ions (Fig. S4b†). These results indicate that the Au and SnO_2_ has been successfully decorated on the surface of SnNbOF.

Besides the Au@SnO_2_ core–shell nanostructure, we also found that the surfaces of the SnNbOF nano-octahedron of the Au–SnNbOF (Au ≥ 3 wt%) were coarse (Fig. S5†) compared with the smooth surface of the blank SnNbOF (Fig. 1c) and the 0.5 wt% Au–SnNbOF (Fig. S6†). The magnified TEM image demonstrates that the SnNbOF surface of the 3 wt% Au–SnNbOF was composed of crystallized SnO_2_ quantum dots with *ca.* 3–4 nm diameters (Fig. S5a and b†). The size of the SnO_2_ dots increased with an increase in Au content (Fig. S5c–f†).

**Fig. 5:**
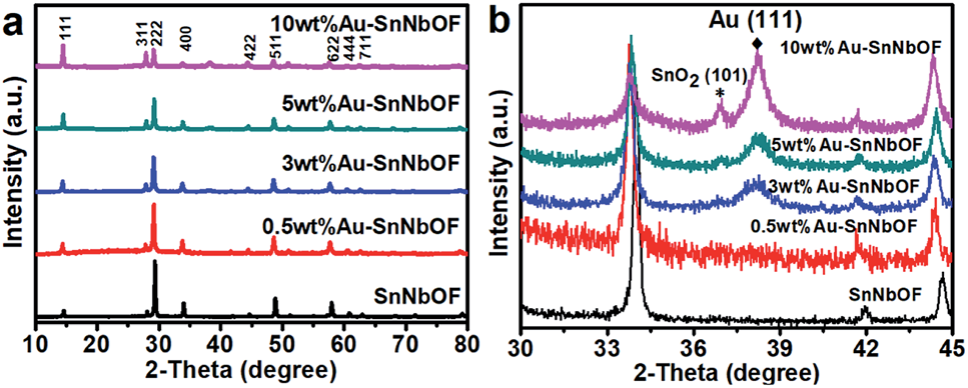
(a) XRD patterns and (b) enlarged XRD patterns of the SnNbOF and the Au–SnNbOF nanocomposites.

Fig. 5a shows the XRD patterns of the SnNbOF and Au–SnNbOF nanocomposites. It is clear that all the Au–SnNbOF nanocomposites, as well as blank SnNbOF, exhibited similar XRD patterns. The main diffraction peaks of all the samples can be attributed to the face-centered cubic crystals of pyrochlore compounds.^28^ The characteristic peaks of SnO_2_ (101)^38^ and Au (111)^39–42^ were also observed at 36.9° and 38.2°, respectively, when the weight ratio of Au was greater than or equal to 3 wt% (Fig. 5b). In contrast, 0.5 wt% Au–SnNbOF exhibited no XRD peaks of SnO_2_ and Au (Fig. 5b).

On comparing the XRD patterns, increasing the Au content was found to result in the following: the intensity of the XRD peaks of the (111) and (311) marked in Fig. 5a progressively increased, and the reflections shifted towards lower 2*θ* angles (Fig. 5b). These phenomena can almost certainly be ascribed to the ion exchange reaction between the Sn^2+^ ions of SnNbOF and the H^+^ ions of HAuCl_4_, which has also been reported in a previous study.^43^ In addition, we also found that intensities of other diffraction peaks of SnNbOF such as (222) and (400) peaks decreased after Au decoration. These results can be attributed to the fact that the relative content of the SnNbOF in Au–SnNbOF nanocomposites decreased when increase the Au content.

The optical properties of the SnNbOF and Au–SnNbOF nanocomposites were investigated *via* UV-vis DRS (Fig. S7†). The blank SnNbOF had visible light absorption capability because of its narrow bandgap (2.33 eV), as reflected in Fig. S8a, b and S9.†^28^ Following Au decoration, a clear absorption peak appeared at *ca.* 550 nm, which can be ascribed to the surface plasmon resonance of Au nanoparticles (Fig. S7†).^44^ In addition, it is also found that the introduction of different Au weight ratios had a significant influence on the optical property of the samples. As shown in Fig. S7,† the absorption intensity within the range of 550–800 nm was enhanced by the increase in the Au weight ratio.

**Fig. 6:**
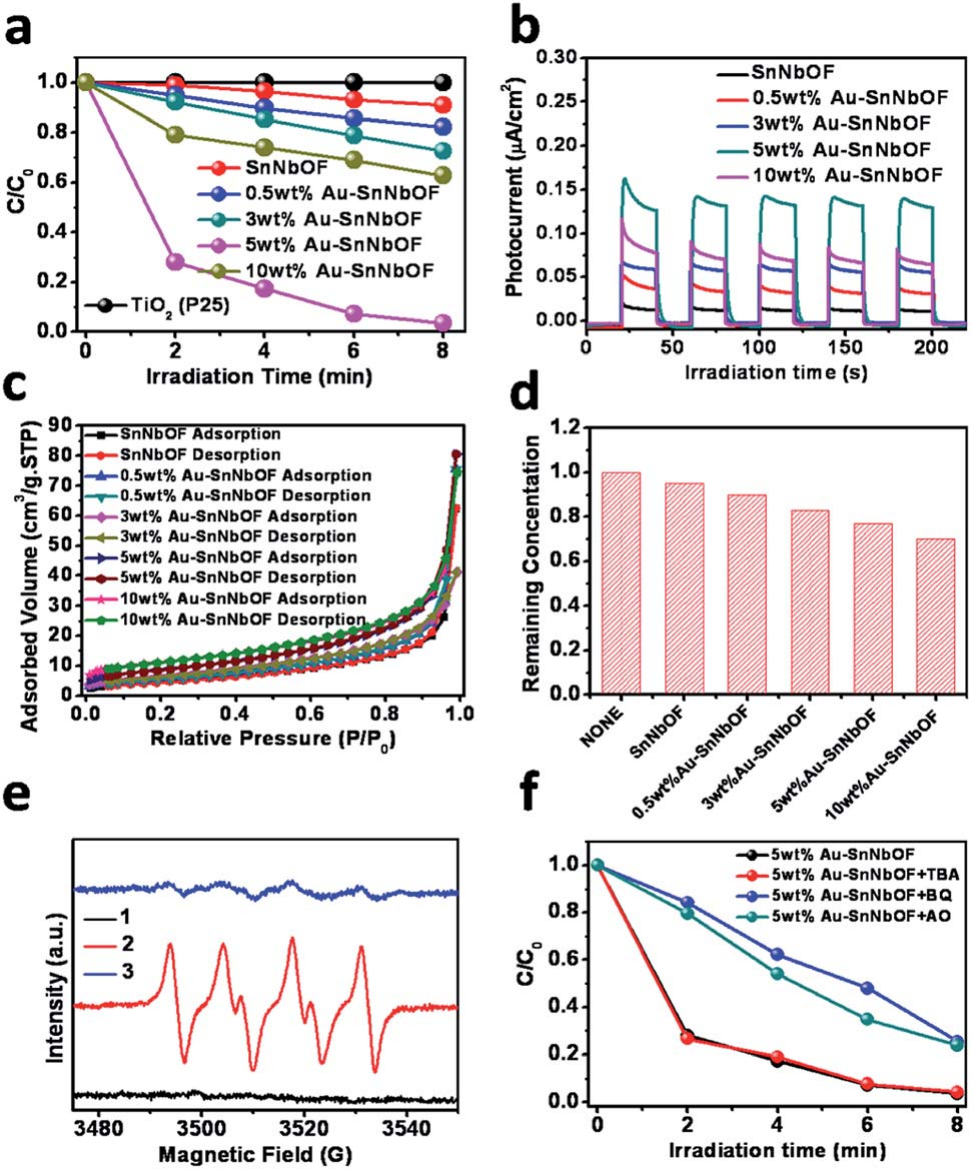
(a) Photocatalytic degradation of MO over the samples under visible light irradiation (420 < *λ* < 800 nm); (b) photocurrent responses of the samples; (c) the N_2_ adsorption–desorption isotherms for the samples; (d) the remaining MO in the solution after reaching the adsorption equilibrium in the dark over the resulting catalyst; (e) DMPO–O_2_^−^˙ formed in the aqueous dispersions of the 5 wt% Au–SnNbOF sample at 77 K under various conditions: (1) dark, (2) under visible light irradiation and (3) after adding MO and (f) photocatalytic degradation of MO in the presence of *tert*-butyl alcohol (TBA), ammonium oxalate (AO) or benzoquinone (BQ) under visible light irradiation over the 5 wt% Au–SnNbOF sample.

In terms of proof of concept, methyl orange (MO) photocatalytic degradation was selected as the probe reaction to demonstrate the application of the samples. As shown in Fig. 6a, the blank SnNbOF exhibited a moderate visible light photoactivity, which was higher than that of TiO_2_ (P25). Meanwhile, the Au–SnNbOF nanocomposites exhibited improved visible light photocatalytic activities than the blank SnNbOF. Among all the photocatalysts, the 5 wt% Au–SnNbOF nanocomposite had the highest rate constant (0.43 min^−1^, Fig. S10†), which was 40 times higher than that of the blank SnNbOF. Within 8 min, the MO was almost completely degraded under visible light irradiation, which significantly exceeded the performance of the SnNbOF. Moreover, the stability of the photocatalyst was also evaluated. As shown in Fig. S11,† following four cycles of photocatalytic reaction, the photocatalytic performance of the used 5 wt% Au–SnNbOF was similar to that of its fresh counterparts. In particular, the activity of the 5 wt% Au–SnNbOF was also compared with the known photocatalysts previously reported, with the photoactivity of the sample found to be superior to most of the reported photocatalysts (Table S2†).

A variety of joint characterization techniques were utilized to reveal the origin of the superior performance of Au–SnNbOF composites for MO degradation. First, the photocurrent response of the samples was measured under visible light irradiation. As shown in Fig. 6b, the photocurrent density obtained across the sample electrodes followed a sequence of 5 wt% Au–SnNbOF > 10 wt% Au–SnNbOF > 3 wt% Au–SnNbOF > 0.5 wt% Au–SnNbOF > SnNbOF. In addition, electrochemical impedance spectroscopy (EIS) Nyquist plots have also been performed (Fig. S12†). The decoration of Au results in obvious decrease of the arc as compared to blank SnNbOF, indicating that the Au–SnNbOF nanocomposites have much smaller charge-transfer resistance than the SnNbOF.^37^ These results suggest a longer lifetime of charge carriers photogenerated over Au–SnNbOF composites than over blank SnNbOF.^37^ This is also supported by the photoluminescence (PL) analysis. As shown in Fig. S13,† the blank SnNbOF exhibits a broad emission peak around 550 nm, which is attributed to the charge recombination. The 5 wt% Au–SnNbOF nanocomposite exhibited obviously reduced intensity of the PL emission of SnNbOF, indicating the reduced charge carriers recombination of Au–SnNbOF nanocomposite in comparison to the blank SnNbOF. This can be ascribed to the fact that the decoration of Au and SnO_2_ on SnNbOF results in an improvement in the charge separation efficiency because of the matched energy band structure (Fig. S14†). Therefore, it would appear that the observed photoactivity sequence could be well correlated with the charge separation efficiency. The highest photoactivity of the 5 wt% Au–SnNbOF was in accordance with its highest charge separation efficiency. Notably, although 10 wt% Au–SnNbOF has the highest amount of Au, its charge separation efficiency is lower than that of 5 wt% Au–SnNbOF. This indicates that the Au amount is an important factor influencing the photoactivity of the sample. At low Au content, Au can act as separation centers and thus improve the photoactivities. However, as Au amount exceed optimum loading, they can act as charge recombination centers, which are detrimental to the photocatalytic efficiency.^31^

Second, we also investigated the influence of the effect of Au nanoparticles on the photoactivities of the samples. Generally, two main mechanisms are proposed to explain the enhanced photoactivity *via* Au nanoparticles, the first of which relates to the plasmon-excited charge transfer from the Au to the semiconductor and the second to the photoexcited electron transfer from the semiconductor to the Au. In the first mechanism, the Au nanoparticles are plasmon-excited under visible light irradiation, meaning the maximum contribution of the Au particles should occur at the strongest plasmon absorption. However, the rate constant of the 5 wt% Au–SnNbOF was very low at 550 nm (Fig. S15†). This indicates that the plasmonic effect of the Au particles was not the main contributor to the enhanced photoactivity. Moreover, the matched energy band structures of SnNbOF and Au are beneficial to the transfer of photo-induced electrons from SnNbOF to Au (Fig. S14†), which could improve the charge separation efficiency, as already revealed by the photocurrent analysis (Fig. 6b). Therefore, it can be stated that Au particles most likely serve as electron collectors to retard the charge recombination.

Finally, the surface area and the adsorption ability of the samples were also investigated. As displayed in Fig. 6c, the nitrogen (N_2_) adsorption–desorption isotherms of the SnNbOF and Au–SnNbOF nanocomposites exhibited type IV isotherms with a typical H3 hysteresis loop characteristic of mesoporous solids.^45^ It is clear from Table S3† that the Au–SnNbOF nanocomposites had larger surface areas than the blank SnNbOF. It is also clear that the surface area of the nanocomposites increased with the increase in Au weight ratio. This increased surface area resulted in enhancing the adsorption capacity of the Au–SnNbOF nanocomposites, as shown in Fig. 6d. The higher photoactivities of the Au–SnNbOF were in accordance with the higher surface area and adsorption capacity. Notably, although the 10 wt% Au–SnNbOF possessed the largest specific surface area and adsorption capacity, its photoactivity was lower than that of the 5 wt% Au–SnNbOF. These results suggest that the primary factor accounting for the photoactivity improvement of Au–SnNbOF nanocomposites cannot be attributed to the differences in specific surface area and the adsorption capacity of the samples; rather, it must be attributed to the improved charge separation efficiency and synergistic interactions among Au, SnO_2_ and SnNbOF.

To study the active radical species involved in the MO degradation, ESR analysis was performed using the 5 wt% Au–SnNbOF sample. Under visible light irradiation, the signal of superoxide radicals (O_2_^−^˙) could be clearly observed, as shown in Fig. S16.†^46^ However, no hydroxyl radicals were detected in the reaction system. These results indicate that O_2_^−^˙ were the primary radical species formed during the reaction. In view of this, the interaction of the O_2_^−^˙ with the reactants was further investigated. As shown in Fig. 6e, under the dark and air atmosphere conditions, no obvious signal was detected. However, when the reaction system was irradiated with visible light, a typical ESR O_2_^−^˙ signal emerged. After adding MO into the reactor, the O_2_^−^˙ signal almost disappeared, indicating that the O_2_^−^˙ were consumed during the photocatalytic reaction.

To get further insight into reaction mechanism for MO degradation, a series of blank/controlled experiments were performed. The blank experiments without catalyst or visible light irradiation show no degradation of MO, which confirms that the reaction is truly driven by a photocatalytic process. As displayed in Fig. 6f, with the addition of benzoquinone (BQ) as superoxide-scavenger,^47^ the photocatalytic reaction became remarkably inhibited. An equally clear inhibition phenomenon within the photocatalytic reaction was also observed when ammonium oxalate was added as hole scavenger.^46^ Moreover, the reaction rate underwent very little change after *tert*-butyl alcohol was added as the hydroxyl radical scavenger,^46^ which is consistent with the ESR result. These results indicate that both superoxide radicals and photogenerated holes are the active species for MO degradation.

Based on these results, a possible reaction mechanism is proposed. Under visible light irradiation, electron–hole pairs are generated from the SnNbOF. The photoexcited electrons are then transferred from the conduction band of the SnNbOF to the Au or SnO_2_ because of the matched energy band structure. Following this, they are captured by the molecular oxygen to form O_2_^−^˙. These active oxygen species are capable of oxidizing MO. Meanwhile, photogenerated holes, which have an anodic potential of 2.1 V *vs.* NHE, are incapable of oxidising H_2_O to form hydroxyl radicals (anodic potential: 2.8 V *vs.* NHE); rather, they directly participate in the MO degradation reaction.

## 4 Conclusions

A simple, green and one-step method was reported in relation to the growth of an Au@SnO_2_ core–shell nanostructure and SnO_2_ quantum dots on SnNbOF nano-octahedron. During this process, SnNbOF acts as a redox-active support for the formation of composite nanostructures. The composition and morphology of the Au–SnNbOF nanocomposites can be simply tuned by varying the weight content of the Au. The Au–SnNbOF hybrids can serve as efficient visible light-driven photocatalysts for MO degradation. The improved charge separation efficiency, increased surface area and adsorption capacity efficiently boost the performance of the hybrids as compared with that of blank SnNbOF.

## Conflicts of interest

There are no conflicts to declare.

## Supplementary Material

RA-010-D0RA06175A-s001
