# Supplementary material for: A redox-active support for the synthesis of Au@SnO2 core–shell nanostructure and SnO2 quantum dots with efficient photoactivities
Source: RSC Adv. 2020 Sep 14;10(56):33955–61. doi: 10.1039/d0ra06175a (PMC9056741; doi:10.1039/d0ra06175a)
Supplement: RA-010-D0RA06175A-s001 [file RA-010-D0RA06175A-s001.pdf]

Electronic Supplementary Material (ESI) for RSC Advances.
This journal is © The Royal Society of Chemistry 2020

Electronic Supplementary Information

A redox-active support for the synthesis of Au@SnO_2 core-shell nanostructure and SnO_2 quantum dots with efficient photoactivities

Xiaoyang Pan,[a*] Wen-Jie Chen,[a] Huizhen Cai,[a] Hui Li,[a] Xue jiao Sun,[a] Bo Weng[b*] and Zhiguo Yi[c*]

[a] *College of Chemistry and Materials, Quanzhou Normal University, Quanzhou, 362000, China.*

[b] *cMACS, Department of Microbial and Molecular Systems, KU Leuven, Celestijnenlaan 200F, 3001 Leuven, Belgium*

[c] *State Key Lab of High Performance Ceramics and Superfine Microstructure, Shanghai Institute of Ceramics, Chinese Academy of Sciences, Shanghai 200050, China.*

**To whom correspondence should be addressed*

E-mail: bo.weng@kuleuven.be; xypan@qztc.edu.cn; zhiguo@mail.sic.ac.cn;

Contents.

Table S1. The reductant, reaction temperature and organic agents involved during the synthesis of Au-metal oxide core-shell nanostructures.

Fig. S1. XRD patterns of SnNbOF.

Fig. S2. The XPS spectra of the SnNbOF: **a**, Survey spectrum; **b**, Sn 3d; **c**, Nb 3d; **d**, O 1s; **e**, F 1s.

Fig. S3. TEM (a) and HRTEM (b) images of Au@SnO_2 core-shell nanostructure of 3wt%Au-SnNbOF.

Fig. S4. The XPS spectra of the 10wt%Au-SnNbOF: **a**, Au 4f; **b**, Sn 3d.

Fig. S5. TEM and HRTEM images of SnO_2 dots of Au-SnNbOF nanocomposites: 3wt%Au-SnNbOF (a, b), 5wt%Au-SnNbOF (c, d) and 10wt%Au-SnNbOF (e, f).

Fig. S6. Additional TEM image of 0.5wt%Au-SnNbOF.

Fig. S7. UV-vis diffuse reflectance spectra of SnNbOF and Au-SnNbOF nanocomposites.

Fig. S8. The plot of transformed Kubelka-Munk function versus the light energy of SnNbOF (a); Mott-Schottky plot of SnNbOF (b).

Fig. S9. Energy band structure of SnNbOF.

Fig. S10. The kinetic rate constant curves of photocatalytic degradation of methyl orange under visible light irradiation over the samples.

Fig. S11. Recycled testing of photocatalytic activity of 5wt%Au-SnNbOF toward the methyl orange degradation under visible light irradiation ($420<\lambda<800$ nm).

Table S2. Comparison of reaction rate constant of 5wt%Au-SnNbOF with those of other Photocatalysts reported in literatures.

Fig. S12. Electrochemical impedance spectroscopy (EIS) Nyquist plots of the sample electrodes of the blank SnNbOF and Au-SnNbOF nanocomposites.

Fig. S13. Photoluminescence spectra of the blank SnNbOF and 5wt%Au-SnNbOF nanocomposite.

Fig. S14. Schematic illustration of the charge transfer process over the Au-SnNbOF nanocomposite.

Fig. S15. Absorption spectrum of 5wt%Au-SnNbOF and action spectrum of MO oxidation on 5wt%Au-SnNbOF.

Table S3. The surface areas of the SnNbOF and Au-SnNbOF nanocomposites.

Fig. S16. ESR spectra for DMPO- $O_2^{-\bullet}$ (a) and DMPO-•OH (b) formed in the aqueous

dispersions of 5wt%Au-SnNbOF.

Table S1. The reductant, reaction temperature and organic agents involved during the synthesis of Au-metal oxide core-shell nanostructures.

NO.	Sample	Temperature/reductant	Organic agents	References
**1**	**Au@SnO_2**	**R.T./NONE**	**NONE**	**This work**
**2**	Au-Cu_2O	30 ^oC/ascorbic acid	sodium dodecyl sulfate	1
**3**	Au-Cu_2O	R.T./formaldehyde	polyvinylpyrrolidone	2
**4**	Au@Cu_2O	0 ^oC /trisodium citrate	polyvinylpyrrolidone	3
**5**	Au@ZnO	95/ citrate	polydiallyldimethylammonium	4
**6**	Au@TiO_2	R.T./trisodium citrate	hydroxypropyl cellulose	5
**7**	Au@Fe_3O_4	95 ^oC / citrate	polyvinylpyrrolidone	6
**8**	Au@TiO_2	180 ^oC /trisodium citrate	sodium citrate	7
**9**	Au@TiO_2	180 ^oC /sodium citrate	sodium citrate and ascorbic acid	8
**10**	Au@SnO_2	850 ^oC /sodium citrate	sodium citrate	9
**11**	Au@SnO_2	60 ^oC/sodium citrate	sodium citrate	10
**12**	Au@CeO_2	160 ^oC /glucose	glucose and urea	11

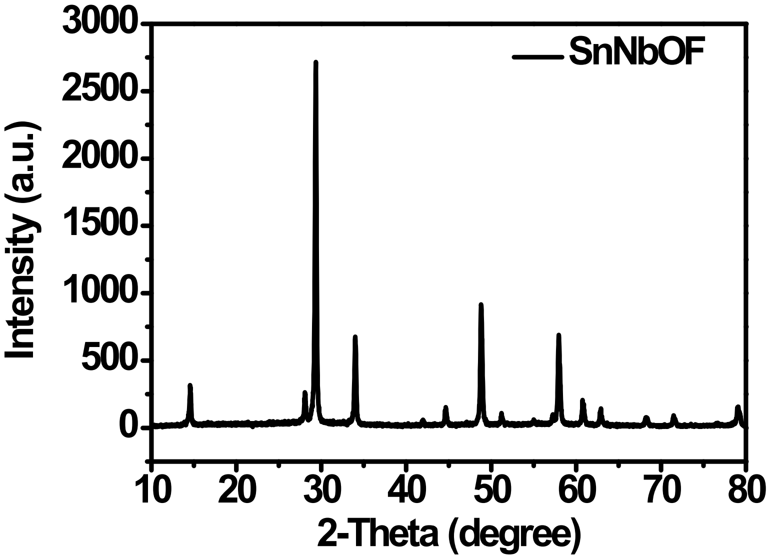

Fig. S1. XRD patterns of SnNbOF.

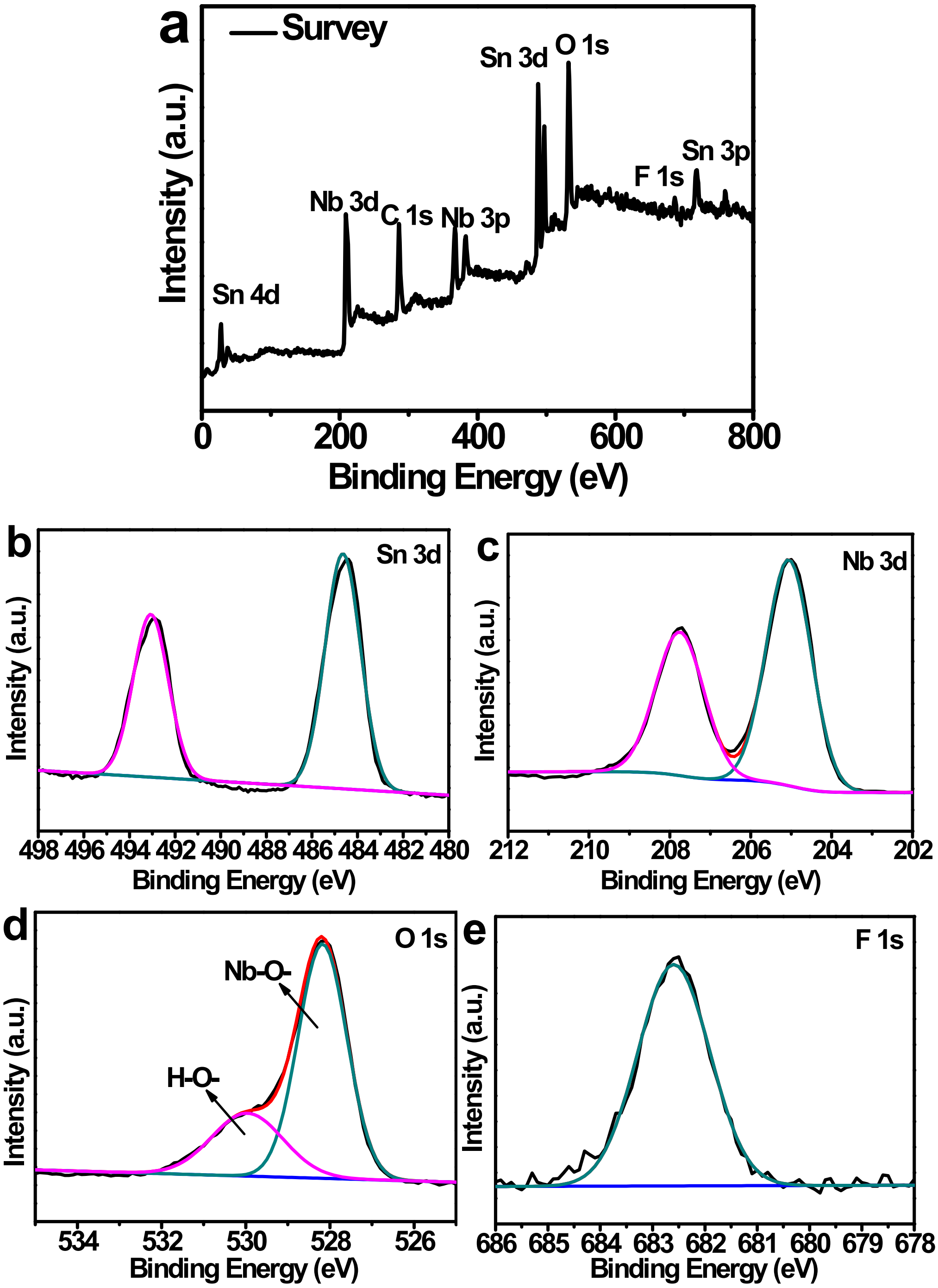

Fig. S2. The XPS spectra of the SnNbOF: **a**, Survey spectrum; **b**, Sn 3d; **c**, Nb 3d; **d**, O 1s; **e**, F 1s.

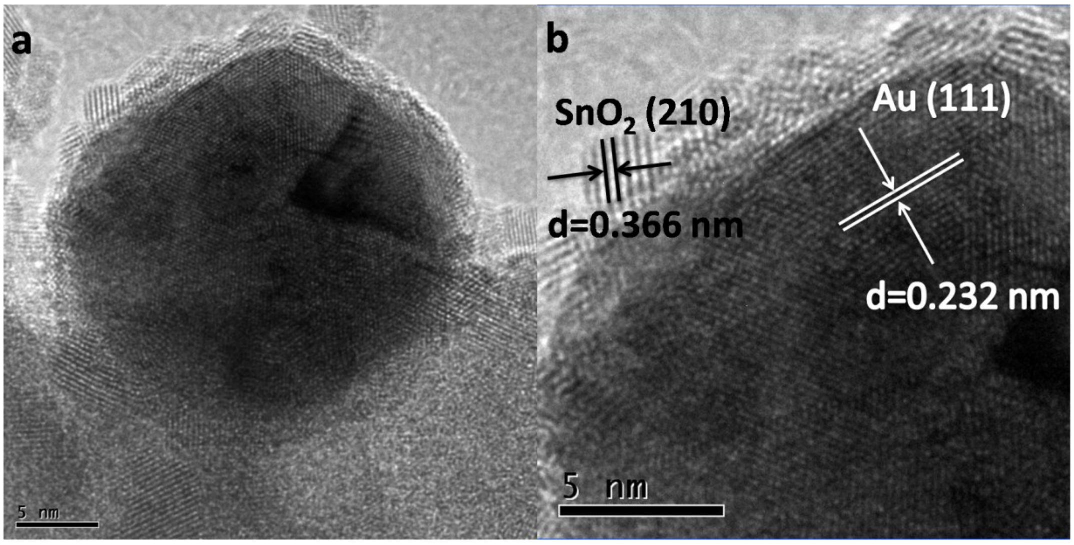

Fig. S3. TEM (a) and HRTEM (b) images of Au@SnO_2 core-shell nanostructure of 3wt%Au-SnNbOF.

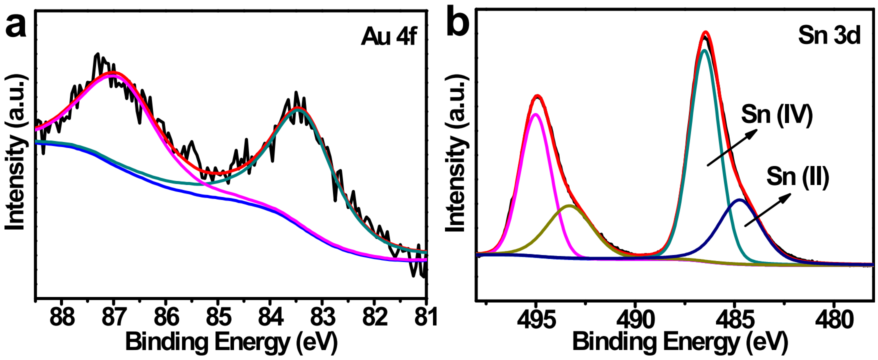

Fig. S4. The XPS spectra of the 10wt%Au-SnNbOF: **a**, Au 4f; **b**, Sn 3d.

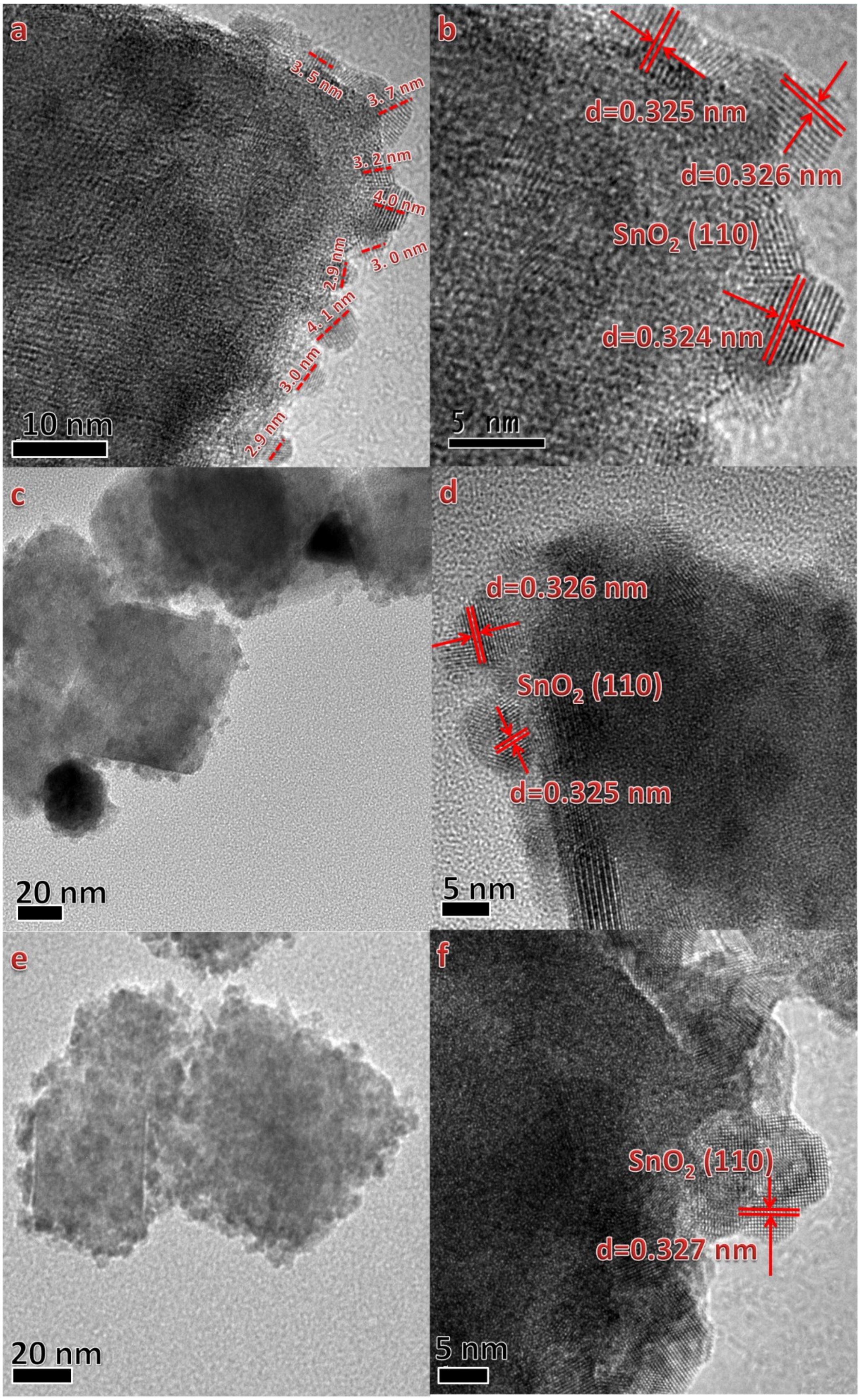

Fig. S5. TEM and HRTEM images of SnO_2 dots of Au-SnNbOF nanocomposites: 3wt%Au-SnNbOF (a, b), 5wt%Au-SnNbOF (c, d) and 10wt%Au-SnNbOF (e, f).

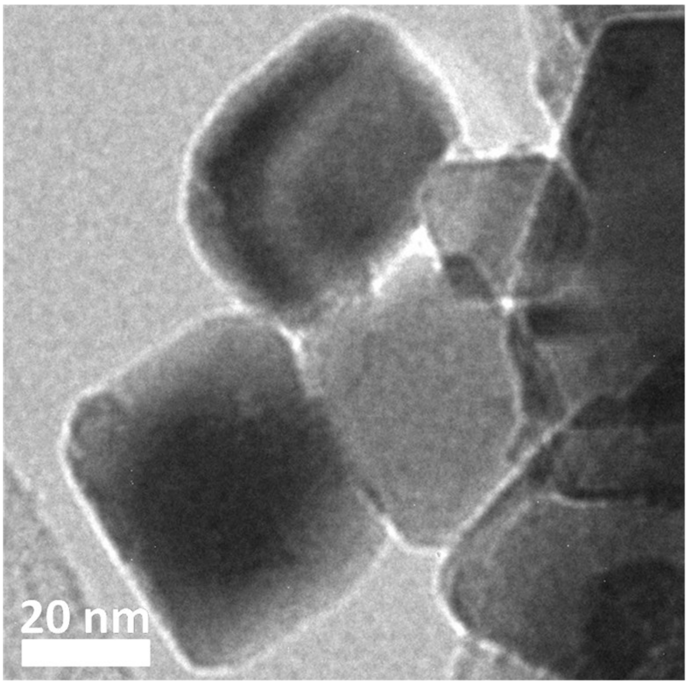

Fig. S6. Additional TEM image of 0.5wt%Au-SnNbOF.

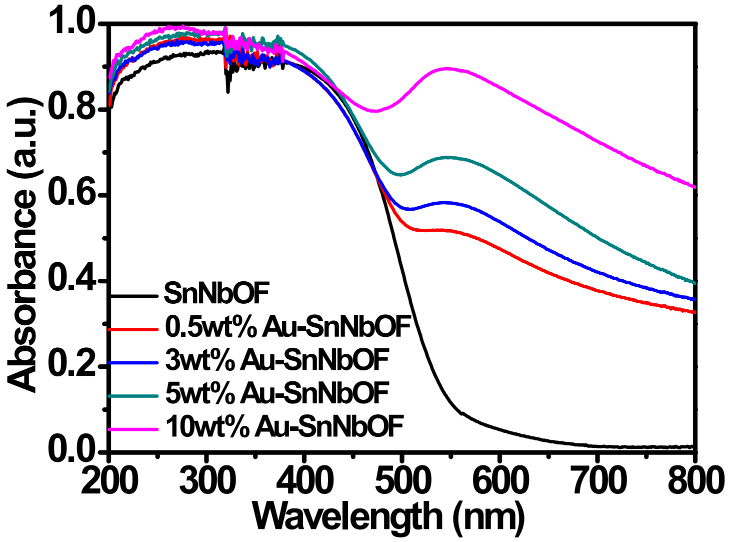

Fig. S7. UV-vis diffuse reflectance spectra of SnNbOF and Au-SnNbOF nanocomposites.

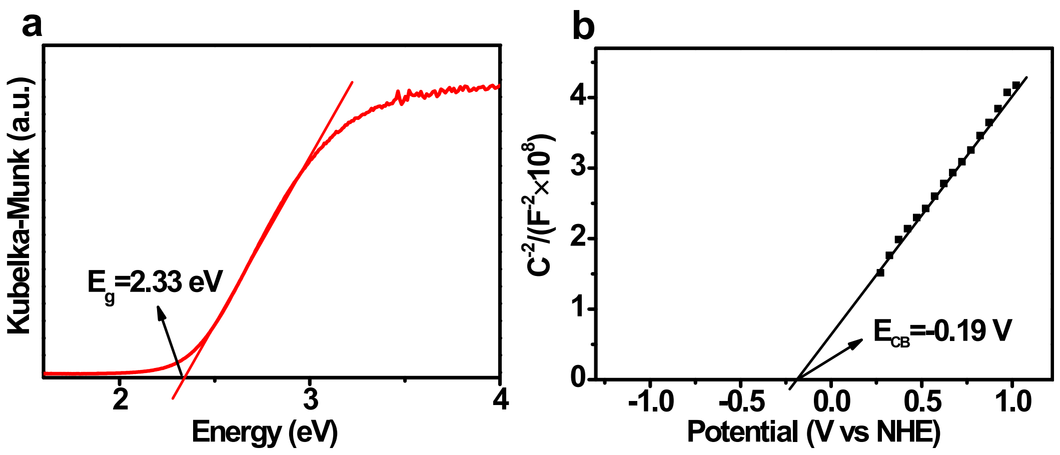

Fig. S8. The plot of transformed Kubelka-Munk function versus the light energy of SnNbOF (a); Mott-Schottky plot of SnNbOF (b).

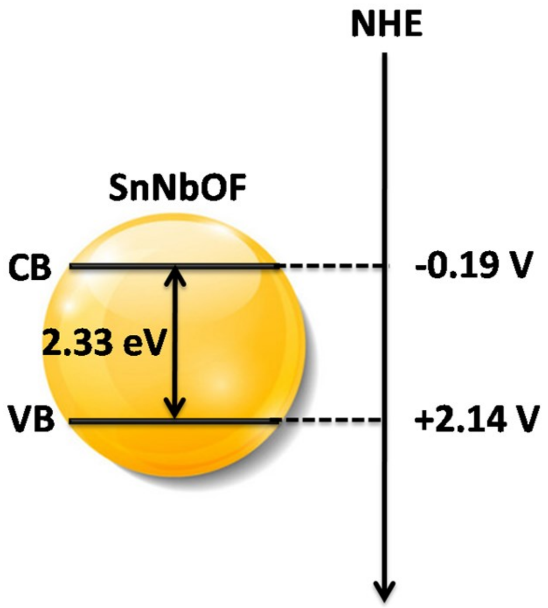

Fig. S9. Energy band structure of SnNbOF.

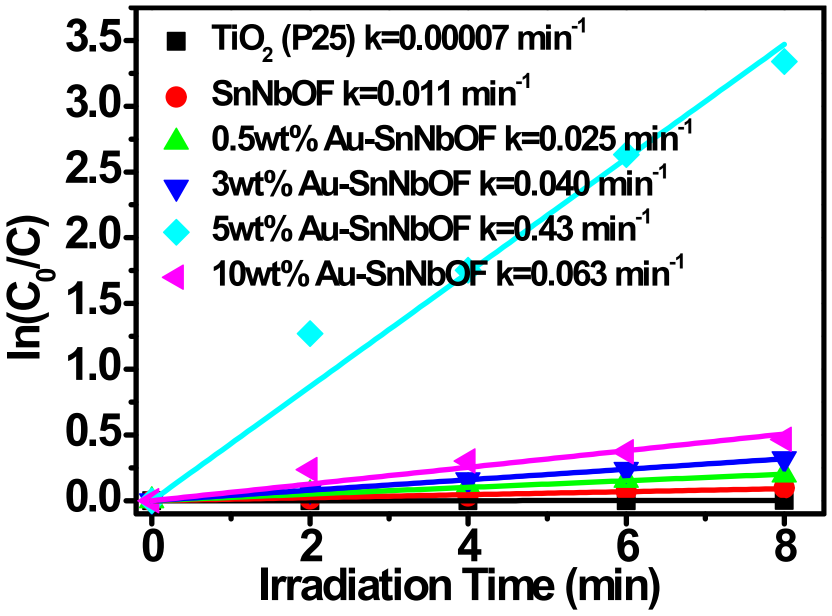

Fig. S10. The kinetic rate constant curves of photocatalytic degradation of methyl orange under visible light irradiation over the samples.

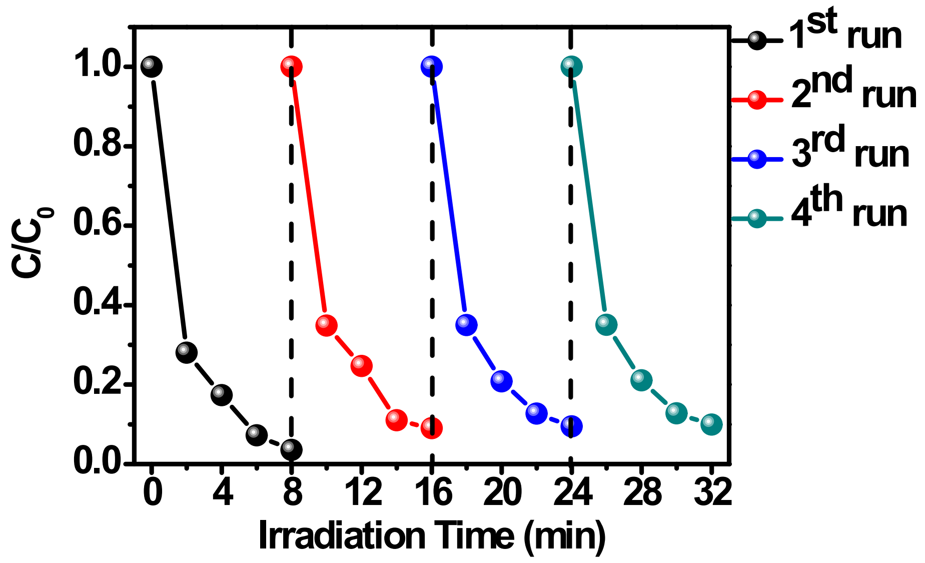

Fig. S11. Recycled testing of photocatalytic activity of 5wt%Au-SnNbOF toward the methyl orange degradation under visible light irradiation (420<λ<800 nm).

Table S2. Comparison of reaction rate constant of 5wt%Au-SnNbOF with those of other photocatalysts reported in literatures.

NO.	Photocatalysts	Rate Constant	Conditions	Literatures
1	5wt%Au-SnNbOF	**0.43 min^{-1}**	30 mg Catalyst MO (60 mL, 10 ppm) visible light irradiation	**This work**
2	95wt%TiO_2/CNT	**0.049 min^{-1}**	80 mg Catalyst MO (80 mL, 10 ppm) UV light irradiation	12
3	P25 (TiO_2)	**0.037 min^{-1}**	80 mg Catalyst MO (80 mL, 10 ppm) UV light irradiation	12
4	3.7%CdS-Bi_2WO_6	**0.019 min^{-1}**	150 mg Catalyst MO (50 mL, 10 ppm) visible light irradiation	13
5	0.2wt%Co_3O_4 / C_3N_4	**0.017 min^{-1}**	100 mg Catalyst MO (100 mL, 10 ppm) visible light irradiation	14
6	70wt% g-C_3N_4/ Bi_2WO_6	**0.037 min^{-1}**	150 mg Catalyst MO (50 mL, 10 ppm) visible light irradiation	15
7	Bi_2WO_6	**0.0008 min^{-1}**	150 mg Catalyst MO (50 mL, 10 ppm) visible light irradiation	15
8	g-C_3N_4	**0.009 min^{-1}**	150 mg Catalyst MO (50 mL, 10 ppm) visible light irradiation	15
9	40%AgBr/WO_3	**0.016 min^{-1}**	100 mg Catalyst MO (100 mL, 10 ppm) visible light irradiation	16
10	TiO_2 nanosheets supported on carbon fibers	**0.058 min^{-1}**	MO (5 ppm) UV light irradiation	17
11	TiO_2 nanosheets supported on FTO glass	**0.017 min^{-1}**	MO (5 ppm) UV light irradiation	17
12	0.06wt%Au-CdS-TiO_2	**0.012 min^{-1}**	150 mg Catalyst MO (100 mL, 10 ppm) visible light irradiation	18
13	CdS-TiO_2	**0.0046 min^{-1}**	150 mg Catalyst MO (100 mL, 10 ppm) visible light irradiation	18
14	SnO_2	**0.0004 min^{-1}**	2 mg Catalyst MO (20 mL, 10 ppm) visible light irradiation	19

15	Ag-SnO_2	**0.004 min^{-1}**	2 mg Catalyst mL, 10 ppm) visible light irradiation	MO (20	19

Note: Only the optimized results of the composite photocatalyst are given in the table.

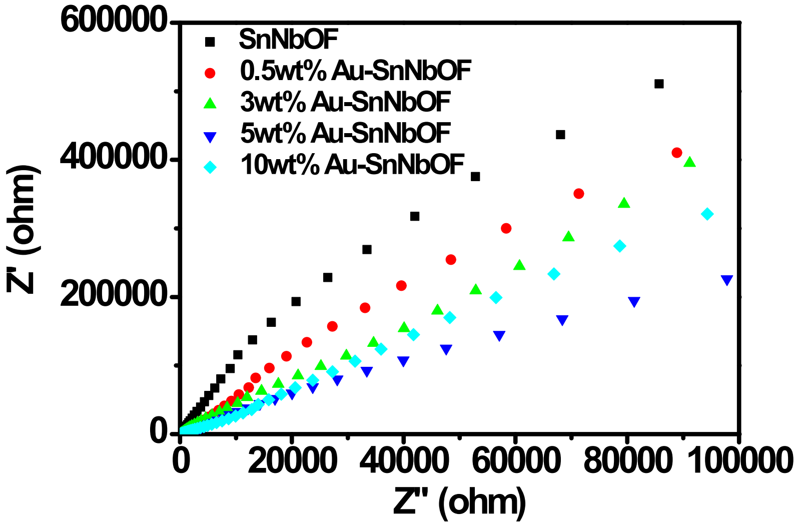

Fig. S12. Electrochemical impedance spectroscopy (EIS) Nyquist plots of the sample electrodes of the blank SnNbOF and Au-SnNbOF nanocomposites.

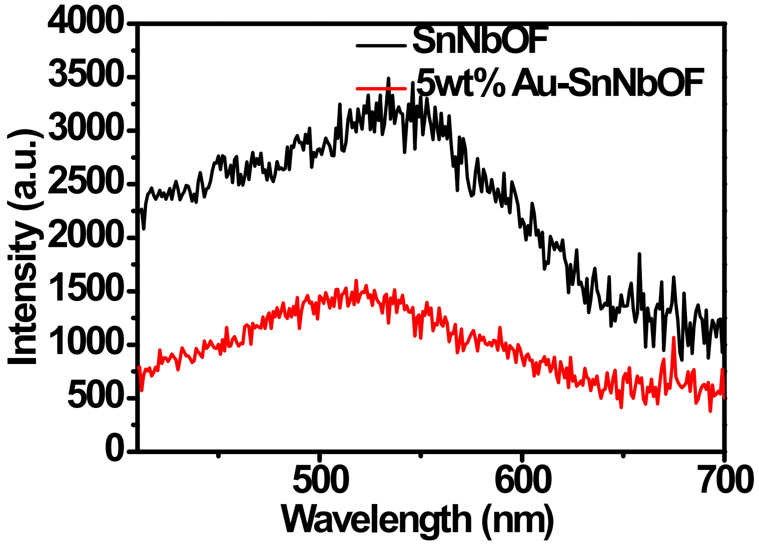

Fig. S13. Photoluminescence spectra of the blank SnNbOF and 5wt%Au-SnNbOF nanocomposite.

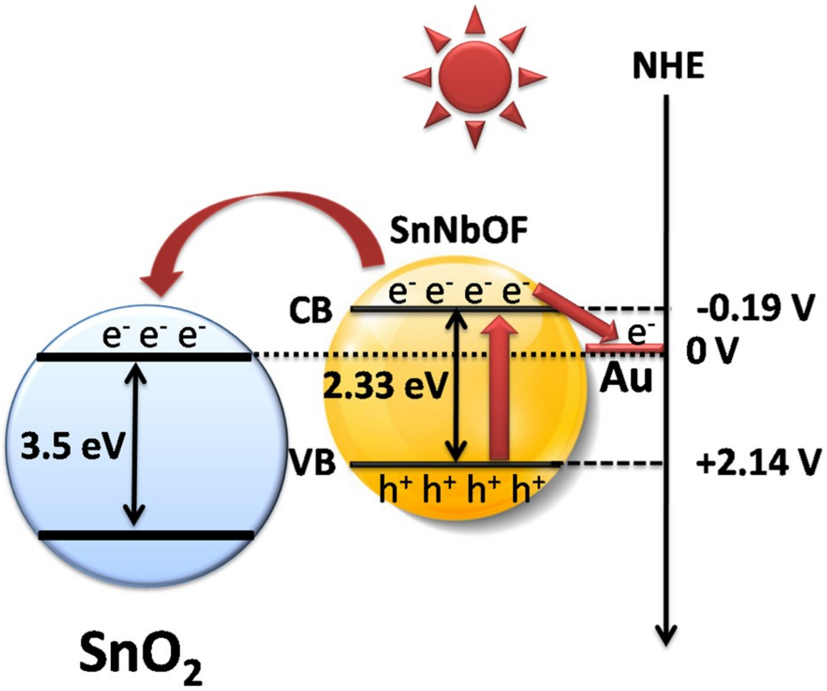

Fig. S14. Schematic illustration of the charge transfer process over the Au-SnNbOF nanocomposite.

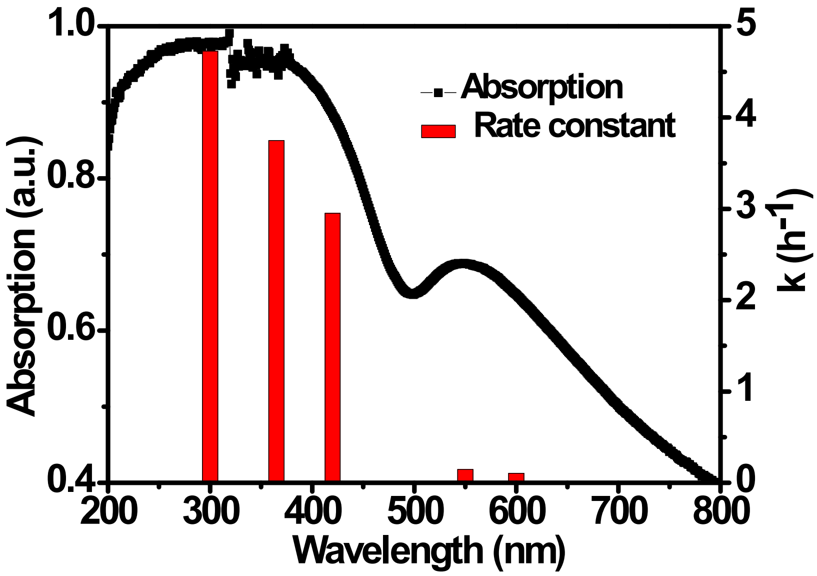

Fig. S15. Absorption spectrum of 5wt%Au-SnNbOF and action spectrum of MO oxidation on 5wt%Au-SnNbOF.

Table S3. The surface areas of the SnNbOF and Au-SnNbOF nanocomposites.

Sample	S_{BET} (m^2/g)
SnNbOF	18
0.5wt%Au-SnNbOF	21
3wt%Au-SnNbOF	25
5wt%Au-SnNbOF	31
10wt%Au-SnNbOF	40

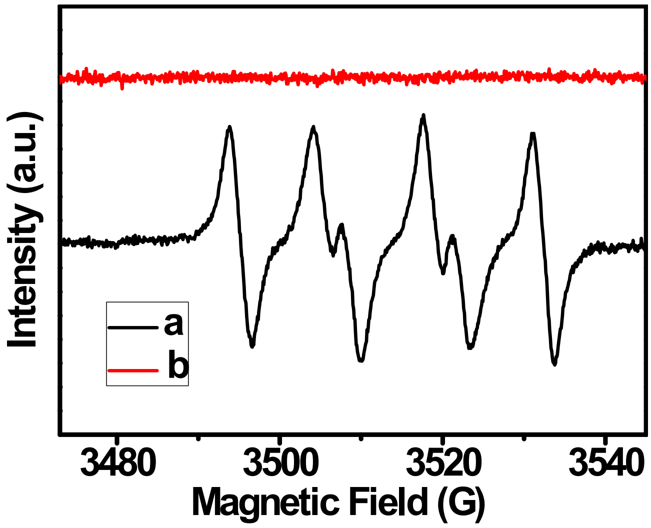

Fig. S16. ESR spectrum for DMPO- $O_2^{-\bullet}$ (a) and DMPO-•OH (b) formed in the aqueous dispersions of 5wt%Au-SnNbOF.

References

1. W.-C. Wang, L.-M. Lyu and M. H. Huang, *Chem. Mater.*, 2011, **23**, 2677.
2. L. Zhang, D. A. Blom and H. Wang, *Chem. Mater.*, 2011, **23**, 4587.
3. D.-Y. Liu, S.-Y. Ding, H.-X. Lin, B.-J. Liu, Z.-Z. Ye, F.-R. Fan, B. Ren and Z.-Q. Tian, *J. Phys. Chem. C*, 2012, **116**, 4477.
4. Y. Sun, Y. Sun, T. Zhang, G. Chen, F. Zhang, D. Liu, W. Cai, Y. Li, X. Yang and C. Li, *Nanoscale*, 2016, **8**, 10774.
5. Z. W. Seh, S. Liu, S.-Y. Zhang, K. W. Shah and M.-Y. Han, *Chem. Commun.*, 2011, **47**, 6689.
6. H. Sun, J. He, J. Wang, S.-Y. Zhang, C. Liu, T. Sritharan, S. Mhaisalkar, M.-Y. Han, D. Wang and H. Chen, *J. Am. Chem. Soc.*, 2013, **135**, 9099.
7. N. Zhang, S. Liu, X. Fu and Y.-J. Xu, *J. Phys. Chem. C*, 2011, **115**, 9136.
8. X.-F. Wu, H.-Y. Song, J.-M. Yoon, Y.-T. Yu and Y.-F. Chen, *Langmuir*, 2009, **25**, 6438.
9. K. Yu, Z. Wu, Q. Zhao, B. Li and Y. Xie, *J. Phys. Chem. C*, 2008, **112**, 2244.
10. G. Oldfield, T. Ung and P. Mulvaney, *Adv. Mater.*, 2000, **12**, 1519.
11. J. Qi, J. Chen, G. Li, S. Li, Y. Gao and Z. Tang, *Energy Environ. Sci.*, 2012, **5**,

8937.
12. Y.-J. Xu, Y. Zhuang and X. Fu, *J. Phys. Chem. C*, 2010, **114**, 2669.
13. L. Ge and J. Liu, *Appl. Catal., B*, 2011, **105**, 289.
14. C. Han, L. Ge, C. Chen, Y. Li, X. Xiao, Y. Zhang and L. Guo, *Appl. Catal., B*, 2014, **147**, 546.
15. L. Ge, C. Han and J. Liu, *Appl. Catal., B*, 2011, **108-109**, 100.
16. J. Cao, B. Luo, H. Lin and S. Chen, *J. Hazard. Mater.*, 2011, **190**, 700.
17. W. Guo, F. Zhang, C. Lin and Z. L. Wang, *Adv. Mater.*, 2012, **24**, 4761.
18. T. Lv, L. Pan, X. Liu and Z. Sun, *Electrochim. Acta*, 2012, **83**, 216.
19. S. A. Ansari, M. M. Khan, M. O. Ansari, J. Lee and M. H. Cho, *RSC Adv.*, 2014, **4**, 26013.
